# Circulating microRNAs and cross sectional and longitudinal measurements of physical functioning and frailty: An explorative study in older twins

**DOI:** 10.1016/j.mad.2025.112099

**Published:** 2025-07-16

**Authors:** Rossella La Grotta, Cecilie Agergaard Sørensen, Asmus Cosmos Skovgaard, Mikael Thinggaard, Serena Dato, Giuseppina Rose, Jonas Mengel-From, Mette Soerensen

**Affiliations:** a Department of Biology, Ecology and Earth Sciences, University of Calabria, Via Pietro Bucci, Rende CS, Calabria 87036, Italy; b Danish Twin Registry, and Research Unit for Epidemiology, Biodemography and Biostatistics, Department of Public Health, University of Southern Denmark, Campus Vej 55, Odense M 5320, Denmark; c Department of Clinical Genetics, Odense University Hospital, Winsløws Vej 4, Odense C 5000, Denmark

**Keywords:** Physical functioning, Activity of daily living, Frailty, Micro RNAs, Monozygotic twins

## Abstract

During aging, physical functioning declines, and disability and frailty increase; phenotypes which are bidirectionally linked. MicroRNAs (miRNAs) are epigenetic regulators of various physiological processes and suggested aging biomarkers. Here we investigate the association between circulating plasma miRNAs and hand grip strength, chair stand, (Rockwood) frailty, and activity of daily living (ADL) in 86 monozygotic twins (73–88 years). In cross-sectional analysis, both individual and twin-pair level analyses were performed, the latter controlling genetic confounding. The majority (74–100 %) of miRNAs identified in the individual-level analysis were validated by twin-pair-level analysis, with 14 miRNAs showing significance (p <0.05) in both. Longitudinal analysis (up to eight years of follow-up) yielded more significant results (75–93 miRNAs), indicating that miRNAs might be more accurate in predicting functional decline over time. Of these miRNAs, seven showed consistent directions of effects across phenotypes. For all analyses, most (65–79 %) of the observed effect sizes were negative, reflecting reduced functionality with increased miRNA levels. Enrichment analyses revealed pathways of gene expression (incl. p53- and FOXO-mediated transcription), signal transduction, the immune system, metabolism of RNA, among others. Of specific miRNAs, miR-1274a demonstrated negative association in both cross-sectional and longitudinal investigations of ADL. These findings support miRNAs as biomarkers of age-related functional decline.

## 1. Introduction

As people age physiologically, their body composition changes (Yu et al., 2014); for instance, the muscle mass decreases by about 8 % every decade from ages 50–70, and by 15 % per decade after age 70 (Grimby and Saltin, 1983). As life expectancy increases, the age-related loss of muscle mass and physical function can lead to more health problems and higher costs for individuals, society, and healthcare systems. Hence, understanding the causes of age-related loss of physical function is crucial for effective prevention, detection, and treatment (Roubenoff, 2000).

Loss of physical function is both a consequence and a contributor to sarcopenia and frailty, in a vicious cycle of functional decline. Sarcopenia is a condition characterized by a decline in muscle quantity and quality, muscle strength, and physical performance and it can occur at any age but is most common among older adults (Cruz-Jentoft et al., 2010). For classification of sarcopenia, data on among others dual-energy x-ray absorptiometry of muscle mass, hand grip strength and chair stand test are used (Cruz-Jentoft et al., 2019). Sarcopenia is, furthermore, a key component of frailty, a condition that affects multiple systems, including the muscular, neurological, cardiovascular and pulmonary systems, and which requires the presence of at least three of the following five criteria, reflecting loss of functional capacity: weakness, slow walking speed, low physical activity, fatigue, and unintentional weight loss (Fried et al., 2001). For systematic estimation of frailty, a number of frailty indexes have been created, including the Rockwood frailty index (Rockwood et al., 2011) reflecting the number of deficits of disease, symptoms, disability, and lack of general wellbeing a person holds. Frailty is considered a multi-domain condition encompassing the social, psychological, cognitive, and physical domains, all of which are interconnected and contribute to the development of chronic age-related diseases (Grande et al., 2019; Maxwell and Wang, 2017). Consequently, frailty increases the risk of disability, admission to nursing homes and mortality (Gordon et al., 2014). Despite both sarcopenia and frailty being categorized as geriatric conditions, they are defined differently, a distinction which suggests that sarcopenia often precedes frailty and contributes to the onset of physical decline (Mori and Tokuda, 2019), and, furthermore, suggests that both traits might share a common biological basis. As a result, early detection of poor functional status in older patients should be a top medical priority (Dent et al., 2019), nevertheless, a biomarker, which may quickly and accurately predict functional impairment is lacking, despite a considerable number of studies of metabolic, microbial, and inflammatory biomolecules (Picca et al., 2020).

MicroRNAs (miRNAs) have been found to be important regulators of physiological and pathological processes and are therefore of particular interest in the diagnosis and treatment of multiple diseases, including sarcopenia (Jung and Suh, 2014) and frailty (Dato et al., 2022). miRNAs are small non-coding RNAs of 18–25 nucleotides in length that can regulate gene expression at the post-transcriptional level (Jung and Suh, 2014). Each miRNA can bind to multiple messenger RNAs (mRNAs) through base pairing, inhibiting their translation or promoting their degradation (Yin et al., 2020). Consequently, miRNAs are involved in biological processes, and several diseases have been linked to their aberrant expression (Yin et al., 2020). In muscle biology, miRNAs play a pivotal role in myogenesis and can affect the regulation of satellite cells, differentiation, and regeneration through their target genes (Yin et al., 2020). Abnormal expression of miRNAs in relation to measures of physical function in older individuals has been analyzed by cross-sectional studies of candidate miRNAs, and several miRNAs have been found to be either up- or downregulated, thus, prompting miRNAs as biomarkers of muscle frailty and sarcopenia (e.g., He et al., 2020; Iannone et al., 2020; Liu et al., 2021; Rusanova et al., 2018; Valášková et al., 2021). However, to the best of our knowledge, only one study has investigated numerous miRNAs found in blood samples in relation to physical functioning; Murabito et al. (2017) investigated hand grip strength in individuals 24–90 years of age, i.e., the study did not have a focus on the loss of functional capacity in old age.

As far as we know, no study has investigated the association between miRNAs and physical functioning in twins. The use of monozygotic (MZ) twins offers a chance to investigate how environmental and genetic factors contribute to the development and progression of physical frailty. Since MZ twins share the same genetic heritage, phenotypic differences within a twin pair can be attributed mainly to non-shared environmental exposures (Tan et al., 2015). Hence, twin analyses exclude the genetic confounding, as well as the confounding introduced by shared early life environmental factors, which often bias molecular epidemiological studies in singletons. This design is also known as the discordant twin pair design, and it is statistically powerful (Li et al., 2018). Here we used this study design to identify associations between miRNAs and parameters of physical function and decline in older twins. To achieve this, we examined the relationship between 265 miRNAs, measured in blood plasma by the Taqman OpenArray approach, and physical function measures in 86 old twins (ages 73–88, with a mean age of 79); we investigated measures of activity of daily living (ADL), and frailty (as estimated by the Rockwood frailty index), as well as hand grip strength, and the 5-times sit-to-stand test. This work included both individual level and intra-twin pair level analyses (i.e., analysis between twins in each pair) to understand the differences between the pairs and to uncover miRNAs that may be influenced by environmental factors. Lastly, we also performed longitudinal analysis of miRNA levels at baseline, and the changes in phenotypes over time, including up to five phenotype measurements over 8 years.

We believe that finding differences in miRNA expression linked to physical function and decline may improve our understanding of the molecular processes that influence muscle performance as we age. This could potentially lead to the development of new methods to monitor physical decline, or to new therapies to halt or reverse the loss of strength and function in older individuals.

## 2. Materials and methods

### 2.1. Study population

The Longitudinal Study of Aging Danish Twins (LSADT), which includes twins aged 70 years or older and living in Denmark at the time of enrolment, provided the data used in the present study. The LSADT was started in 1995 and continued every second year until 2005 (Pedersen et al., 2019). In 2007, the follow-up was extended to all twin pairs where both co-twins were still alive. Twin zygosity was classified through questionnaire data, a method which correctly classify > 95 % of the twin pairs (Christiansen et al., 2003). Blood samples were taken from 698 individuals in 1997 and from 121 individuals in 2007 and kept at −80°C in RNase-free tubes. All participants underwent comprehensive interview-based questionnaires and examinations, designed to evaluate among others cognitive status, functional abilities, physical health, and social aspects. Informed consent was obtained from all participants, and the survey was approved by the Regional Scientific Ethical Committees for Southern Denmark (S-VF-20040241) and conducted in accordance with the Helsinki II declaration.

From the LSADT 1997 wave, blood samples from 45 randomly chosen monozygotic (MZ) twin pairs were used to generate data on plasma circulating miRNAs (see Mengel-From et al., 2018 for details). For the present study, we included 86 MZ twins (43 twin pairs) aged between 73 and 88 years (mean age 79.05 ± 3.51 years), of whom 66 were females (mean age 79.15 ± 3.6 years) and 20 were males (mean age 83.73 ± 3.27 years). Participants were eligible if they had both circulating plasma miRNA data and data on physical functioning and functional capacity, including hand grip strength, 5-times sit to stand test and ADL, and frailty. For these individuals, their sex was defined as the sex assigned at birth, as defined in the Danish Civil Registration System Registry (information based on midwife journals). We did not perform sex stratified analysis as the sample sizes would not have enabled a comprehensive analysis.

### 2.2. Assessment of physical performance, functional status, and frailty

#### 2.2.1. Hand grip strength

In the LSADT 1999 wave, and in subsequent waves, the hand grip strength was measured in kilograms (kg) using a handheld dynamometer (SMEDLEY’s dynamometer, Scandidact, Kvistgaard, Denmark), while the subject was sitting with the arm close to his/her body and he/she was asked to squeeze as hard as possible and to perform 3 repetitions with each hand (Frederiksen et al., 2006). The hand grip strength value used in the present study was the maximum value obtained with the strongest hand, for which all participants had made three attempts. To ensure normality of the hand grip strength data, it was log transformed before data analysis.

#### 2.2.2. Chair stand test (5-times sit-to-stand)

In the LSADT 1999 wave, and subsequent waves, the 5-times sit-to-stand test (hereon called chair stand test) was performed: participants were asked to rise from a chair 5 times in a row without pausing and as quick as possible. Participants were asked to keep their arms folded across the chest. The time required was measured in seconds with a stopwatch. To ensure the same direction of effect of all phenotypes, i.e., increasing values meaning better function, the time was reversed before statistical analysis by subtracting the values from the maximum value plus 1.

#### 2.2.3. Functional capacity measured by Activities of Daily Living (ADL)

##### 2.2.3.1. ADL fatigue score.

In the LSADT 1997 wave, and subsequent waves, an ADL Fatigue score was calculated; it assesses how tired a person feels when performing common daily activities and it is calculated as the sum of six ADL items; getting up from a chair/bed, walking around in the house, walking up the stairs, getting outdoors, and taking an outdoor walk in fine or bad weather (Avlund et al., 1996). The six items had a score of 0 or 1, where 0 means the item makes you tired and 1 means it does not. Therefore, the score can range from 0 to 6, with a higher number indicating fewer fatigue problems.

##### 2.2.3.2. ADL strength score.

In the LSADT 1997 wave, and subsequent waves, an ADL strength score was generated: it consists of 11 questions that relate to an individual’s ability to perform daily physical activities such as walking around the house, climbing, and descending stairs, walking for 30 min without resting and exercise activities. A score was given as a 1–4 to each question, and the ADL score refers to the average of the 11 items, with higher scores indicating lower levels of strength. If an item was missing or skipped, the average of that item was substituted. If more than one item was skipped, the scale was coded as missing (Christensen et al., 2000). The ADL strength score was also reversed by subtracting the values from 5 (i.e., the maximum value plus 1).

##### 2.2.3.3. ADL endurance score.

In the LSADT 1999 wave, and subsequent waves, an ADL endurance score was calculated as the average of 3 individual items measuring how long a person can walk, run, and bike without resting. Each item has a score between 1 and 7, where a higher value indicates worse functionality. If one item was absent, the mean was substituted, and the scale was absent if two or more items were omitted. The ADL Endurance score was also reversed by subtracting the values from 8 (i.e., the maximum value plus 1).

#### 2.2.4. The frailty index

Based on survey data from the LSADT 1997 wave, a frailty index was calculated. We used the 43-item frailty index, as described in Mak et al. (2024); it was calculated according to the standard method by counting the number of deficits, including diseases, signs, symptoms, and physical functioning, and dividing that number by the total number of deficits considered (Rockwood et al., 2011), i.e., the index reflects the percentage of deficits which a person holds. The index was used as a continuous variable in the main analysis. The index was also reversed by subtracting the values from 1, hence reflecting the percentage deficits a person does not hold. The frailty index was also calculated in all the subsequent waves.

### 2.3. RNA extraction and quantification of miRNA

Circulating miRNAs were extracted from 100 μl blood plasma and quantified by TaqMan-based semi quantitative real-time PCR (qRT-PCR) technology in the autumn of 2015 at the Department of Clinical Biochemistry and Pharmacology at Odense University Hospital (see Mengel-From et al., 2018 for details). In short, the TaqMan OpenArray Human microRNA panel (Life Technologies) covers 377 miRNAs and quantity their expression in for instance a blood sample. A pre-amplification step was followed by a qRT-PCR step, both were carried out in two separate reactions (pools A and B) before quantification cycle values were obtained. These values were used for relative quantification, and finally, normalization using the qBase software (Biogazelle, NV, Belgium), which among others access the stability and quality of reference miRNAs used for normalization (Hellemans and Vandesompele, 2014). Normalization was performed by dividing the relative quantities by a sample specific normalization factor, which is calculated by taking the geometric mean of the relative quantities of selected reference miRNAs (Hellemans et al., 2017) (see Mengel-From et al., 2018 for details). The twin pairs were run in the same qRT-PCR panel. In the 86 participants of the present study, data were available for 265 miRNAs, which had measurement values in minimum 10 of the 86 individuals, similar to (Mengel-From et al., 2018).

### 2.4. Statistical analysis

All statistical analyses were performed using STATA 17.0 (StataCorp; 2021). As mentioned above (Section 2.2) four of the six phenotypes were reversed so the direction of effect was the same, i.e., for all phenotypes the higher the value the better the function. Consequently, in all the analyses a positive beta coefficient corresponds to increased functionality with increasing miRNA level, while a negative beta coefficient corresponds to decreased functionality with increasing miRNA level. We defined statistical significance as P < 0.05.

#### 2.4.1. Cross-sectional analysis

##### 2.4.1.1. Individual level analysis.

A cross-sectional analysis at the individual level was performed using a linear regression model (the regression() function); hand grip strength, chair stand test, ADL, and frailty respectively, were used as outcome and the individual miRNA as exposure. A minimum sample size of 10 individuals was set for the individual level analysis. Sex, age in 1997, and body mass index (BMI) in 1997 were used as covariates, with height in 1997 additionally included for hand grip strength. Data on height and weight were available as self-report. To account for dependency between twins in a pair, we used the Hubert-White-Sandwich (robust) estimator of variance, assuming independence between twin pairs (cluster function).

##### 2.4.1.2. Twin pair level analysis.

Linear regression analysis was performed using the xtreg function with the fe option in STATA, which fits fixed effects models using a within twin pair regression estimator. This model investigates the intra-pair differences with the hand grip strength, chair stand, ADL, and frailty respectively, as outcome, and the individual miRNAs as exposure. Hence, discordance in the present study is simply defined as being different within a twin pair in the phenotypes investigated. A minimum sample size of 5 twin pairs was set for the twin pair analysis (similar to Mengel-From et al., 2018). The models were similar to the individual level analysis adjusted for age, sex, and BMI, with height also included as a covariate in the hand grip strength analysis. However, this intra-pair twin analysis also controls for the confounding introduced by genetic and early life environmental factors, which the individual level analysis does not. Hence, the miRNAs identified in the individual level analysis and confirmed in the twin pair level analysis can be considered the most interesting candidates, when investigating the association between variations in miRNAs and a given phenotype of interest, as they demonstrate a more robust association.

#### 2.4.2. Longitudinal analyses

A longitudinal analysis was performed for the ADL fatigue, ADL strength and frailty scores by using the survey data in the waves 1997, 1999, 2001, 2003 and 2005, and for the hand grip strength, chair stand test and ADL endurance scores using the survey data in the waves 1999, 2001, 2003 and 2005.

A linear mixed-effects model (mixed command in Stata) was used with the phenotype of interest as outcome and the age at interview as exposure. Whether a given miRNA is associated to the change in phenotype over age (i.e., survey waves) was investigated by inclusion of an interaction term between a given miRNA and the age at interview. The same covariates as described above (Section 2.4.1.1) were included. This longitudinal analysis was conducted at the individual level only, with a minimum sample size of 10 individuals, similar to (Mengel-From et al., 2018). Lastly, for description of the longitudinal changes in the phenotypes, a linear mixed-effects model (as described above) was used yet excluding miRNAs and the estimate for age was reported.

### 2.5. Pathway analysis

For investigation of the biological functions of the identified miRNAs, miRNA-target enrichment analysis was conducted using a target prediction tool called MIENTURNET, which applies a set of experimentally validated miRNA-target interactions derived from the miRTarBase database (Licursi et al., 2019). The significance threshold of false discovery rate (FDR) was set to 0.15 (default) for the targets to be investigated in the database, and p value < 0.05 for the miRNA to be used an input. A minimum of 2 gene-miRNA interaction was used. A network of miRNA-target interactions identified in the analysis was constructed in which the most intriguing miRNAs (i.e., the hubs in the network) were submitted for functional enrichment analysis by the REACTOME database, that is embedded within the miRTarBase. Lastly, the pathways identified were grouped by their hierarchical group as defined by the Reactome database.

## 3. Results

In the present study, we investigated 265 circulating miRNAs in 86 individuals from 43 twin pairs. Sample characteristics are shown in Table 1. As indicated, the study population included more females than males, and according to the frailty index, 84 of the 86 individuals were classified as non-frail or pre-frail. To explore associations between miRNAs levels and physical indicators and frailty measures, we performed linear regression analyses at both the individual level and within twin pairs (intra-pair analysis).

### 3.1. Cross sectional analyses at the individual and twin pair levels

#### 3.1.1. Association between miRNAs and hand grip strength

At the individual level, 17 miRNAs were significantly associated with grip strength (p < 0.05), while the twin pair level analysis identified 15 miRNAs (see Supplementary Table 1 for full results). Of the 17 miRNAs from the individual level analysis, 9 had sufficient data (from more than 5 twin pairs) for inclusion in the intra-pair analysis, and all 9 (100 %) displayed consistent directions of effect in both analyses, 6 with negative and 3 with positive beta coefficients. Notably, as shown in Table 2A, miR-625 was significant (p < 0.05) in both models. Its positive regression coefficient suggests that a higher expression is associated with stronger grip strength, indicating improved physical function with increasing miR-625 levels.

#### 3.1.2. Association between miRNAs and chair stand

At the individual level, 25 miRNAs were significantly associated (p < 0.05), while the twin pair level analysis revealed 7 miRNAs (Supplementary Table 2). Of the 25 miRNAs, 20 could be included in the twin pair analysis, and 17 (85 %) exhibited the same direction of effect: 6 with negative and 11 with positive beta coefficients. Notably, as shown in Table 2B, miR-27b and miR-339–5p were statistically significant (p < 0.05) in both models. The negative beta coefficients suggest that as miRNA levels increase, chair-stand test performance worsens - that is, the time to complete the test increases - indicating reduced physical functionality.

#### 3.1.3. Association between miRNAs and measures of Activities of Daily Living

For the ADL fatigue score, 16 and 11 miRNAs were found significant in individual and twin pair level analysis, respectively (Supplementary Table 3). Of the first 16, 10 miRNAs were analyzed at the twin pair level and of these 9 (90 %) showed the same direction of effect (all with negative beta coefficients). Notably, miR-30b and miR-628_5p were found to be significantly reduced in both analyses (Table 2C), hence with a direction of effect reflecting a decrease in the fatigue score per increase in miRNA.

With respect to the ADL strength score, 25 miRNAs were significantly associated with the phenotype in the individual level and 10 in the twin pair level analysis (see Supplementary Table 4). Of the former 25 miRNAs, 23 had sufficient data for inclusion in the twin pair level analysis. Among these, 22 (96 %) showed the same direction of effects (13 with negative beta coefficients and 9 with positive beta coefficients). Among the identified miRNAs, four showed significant associations with the ADL strength score in both the individual- and twin pair level analyses (Table 2C). Specifically, miR-766 and miR-15b were associated with a decrease in strength score as their expression levels increased, indicating reduced physical function. In contrast, miR-454 and miR-518d were linked to an increase in strength score with higher expression level, suggesting improved physical function.

The ADL endurance score was significantly correlated with 38 miRNAs at the individual level and 12 at the twin pair level (Supplementary Table 5). Of the 38 miRNAs from the individual level analysis, 31 were also included in the twin pair analysis, with 23 (74 %) showing consistent directions of effect - 19 with negative and 4 with positive beta coefficients. Notably, miR-601, miR-1274a, and miR-720 emerged as significant in both analyses (Table 2C) all showing negative beta coefficients, indicating reduced endurance function with increasing miRNA expression.

Finally, when comparing miRNAs significantly associated across the three ADL measures within each analysis level, several overlaps emerged (Supplementary Table 6). In the individual level analysis, miR-24 was significantly associated with all three ADL scores, with negative beta coefficients reflecting a decreased functionality with increasing miRNA level. Similarly, in the twin pair level analysis, miR-601 was significantly associated with all three ADLs, also with negative coefficients (Supplementary Table 7). Both miRNAs showed consistent directions of effect across analyses, although not always reaching significance

#### 3.1.4. Association between miRNAs and the frailty index

Finally, when we investigated the miRNA associations with frailty, 26 miRNAs were significantly associated at the individual level and 17 at the twin pair level (Supplementary Table 8). Of the 26 miRNAs identified in the individual level analysis, 22 were included in the twin pair analysis, and 17 of these (77 %) showed consistent directions of effect (11 with negative and 6 with positive beta coefficients). Notably, miR-423–5p and miR-221 were significant in both analyses (Table 2D). Their negative beta value indicates that higher miRNA expression is associated with lower frailty scores suggesting increased frailty (i.e., an increased number of deficits) with increasing miRNA levels.

Furthermore, several miRNAs significantly associated with the frailty index in both analytical approaches, were also linked to other physical function measures (Supplementary Tables 6 and 7). At the individual level, 13 frailty-associated miRNAs were also significant for ADLs and chair stand test; of these, 7 showed consistent directions of effect (miR-221, miR-24, miR-27a, miR-30c, miR-331, miR-656 with β<0, and miR-363 with β>1). In the twin pair level analysis, 6 miRNAs were significantly associated with frailty, ADLs, and hand grip strength, including miR-518d, which consistently showed a positive beta coefficient. These overlapping associations suggest a shared biology across frailty and functional phenotypes, which may also reflect the inclusion of ADL items in the frailty index.

#### 3.1.5. Pathway analysis of the miRNAs identified in the cross-sectional analyses

To explore the biological functions of the 14 miRNAs identified in the cross-sectional analyses (see Table 2), we performed a miRNA-target enrichment analysis using the MIENTURNET prediction tool. To better understand how miRNAs can contribute to either improved or reduced physical function, we grouped them based on the direction of their beta coefficients: mir454, mir518d and mir625 had positive beta coefficients (indicating improved function), while the remaining 11 miRNAs, mir15b, mir1274a, mir221, mir27b, mir30b, mir339_5p, mir423_5p, mir601, mir628_5p, mir720 and, mir766 had negative beta coefficients (indicating reduced function). For the three miRNAs with positive beta coefficients, 8 target genes were significantly enriched (p < 0.05; Supplementary Table 9), while 244 genes were identified for the 11 miRNAs with negative coefficients (Supplementary Table 10).

**Fig. 1.**
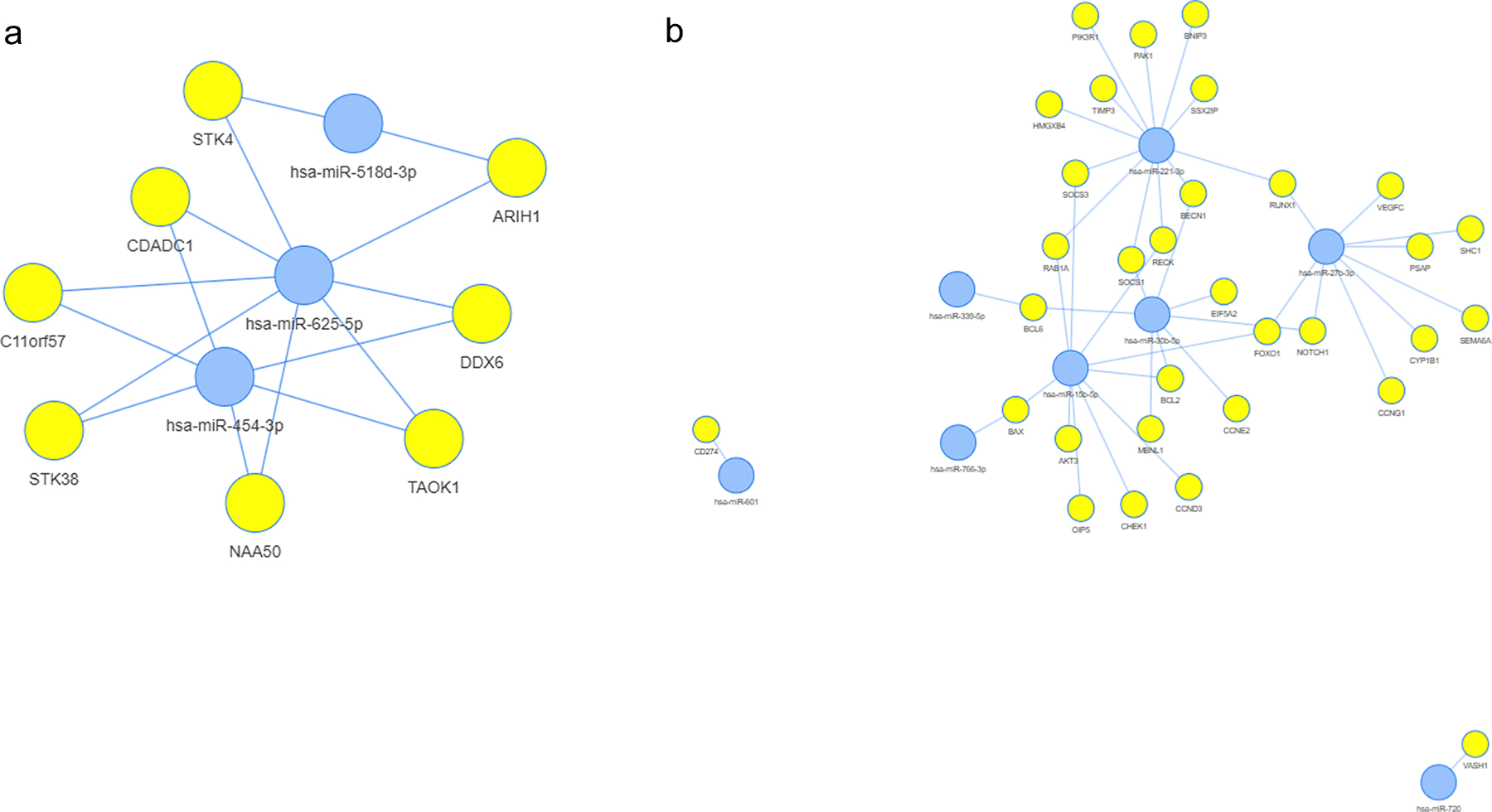
Networks obtained by miRNA-target enrichment analysis (using MIENTURNET) for miRNAs identified in the cross-sectional analyses. (a) The network for the three miRNAs found to display a positive beta coefficient in the cross-sectional analyses. (b) The network for the 11 miRNAs found to display a negative beta coefficient in the cross-sectional analyses.

**Fig. 2.**
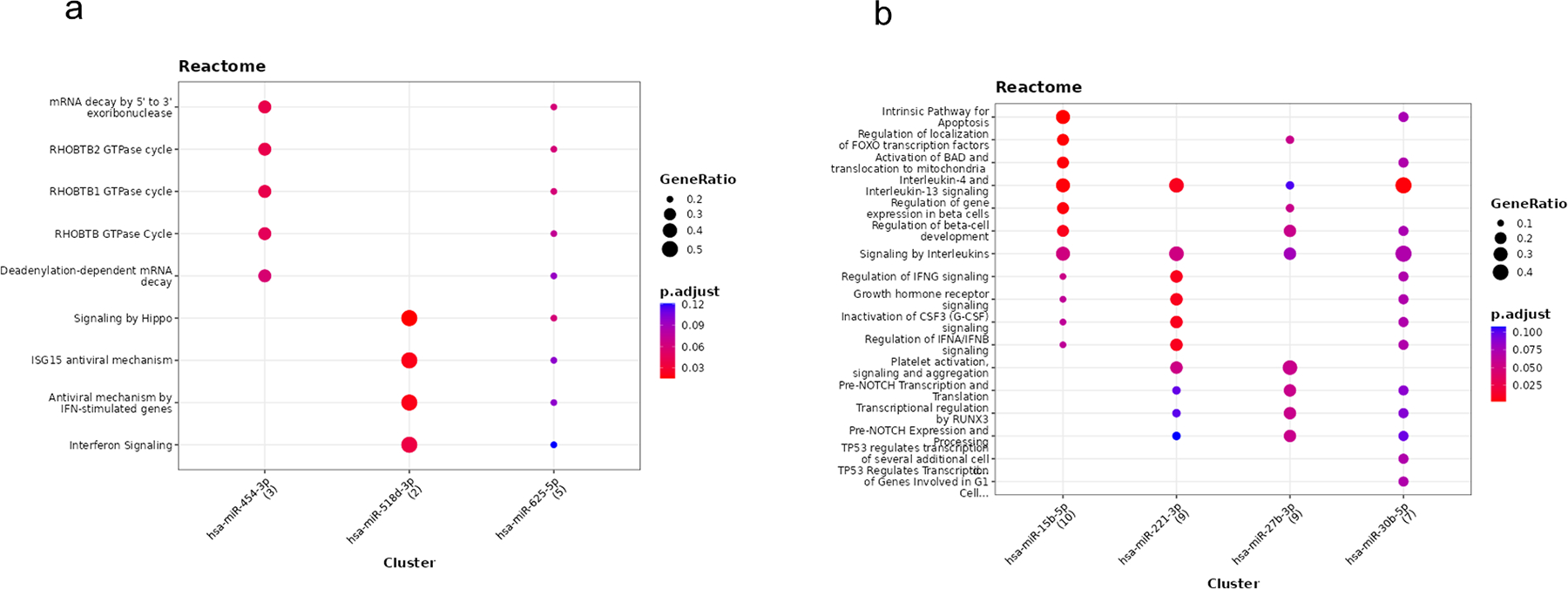
Pathways identified for the miRNAs identified via network analysis (using MIENTURNET) for miRNAs identified in the cross-sectional analyses. (a) Pathway analysis by the Reactome database of the three miRNAs found to display a positive beta coefficient in the cross-sectional analyses. (b) Pathway analysis by the Reactome database of the four miRNAs found as central hub in the network of the 11 miRNAs found to display a negative coefficient in the cross-sectional analyses. Notes: the dots are scaled by the ratio of enriched miRNA targets to the total genes in each pathway. Dot color reflects the adjusted p-value, enabling simultaneous visualization of enrichment magnitude and statistical significance across categories.

The miRNA-target interaction networks generated by the enrichment analysis (Fig. 1a and b) helped us to select the most relevant miRNAs for the functional enrichment analysis by REACTOME. These included miR-454–3p, mR-625–5p and miR-5184–3p (positive beta, Fig. 1a) and miR-15b, miR-221–3p, miR-27b, and miR-30b (negative, Fig. 1b). The pathway analysis showed that miRNAs linked to improved function were involved in the immune system, signal transduction, and RNA metabolism (Fig. 2a and Supplementary Table 11). In contrast, those linked to reduced function were associated with pathways related to the immune system, signal transduction, gene expression, programmed cell death, hemostasis, and developmental biology (Fig. 2b and Supplementary Table 11).

### 3.2. Longitudinal analyses

Firstly, we investigated the overall change in the phenotypes over time in our cohort, which revealed the expected trend, namely a decline in function: hand grip strength (log transformed): coef. = −0.016, p = 5.92*10^−4^, chair stand (reversed): coef. = −0.223, p = 0.12, ADL fatigue: coef. = −0.132, p = 5.11*10^−7^, ADL strength (reversed): coef. = −0.07, p < 1*10^−334^, ADL endurance (reversed): Coef. = −0.115, p = 10^−334^ and Frailty index (reversed): −0.005, p = 1.38*10^−4^. Subsequently, we evaluated the association between baseline miRNA profiles and longitudinal changes in phenotypic expression.

#### 3.2.1. Longitudinal analysis of baseline miRNA levels and phenotypic decline over time

Eighty-seven miRNAs were found associated (p < 0.05) with hand grip strength, 75 miRNAs with chair stand test, 88 miRNAs with ADL strength score, 82 miRNAs with ADL Fatigue score, 78 miRNAs with ADL Endurance score and 93 miRNAs with Frailty score (Supplementary Table 12). Twenty-five miRNAs were especially notable, with p-values < 0.05 in most phenotypes (4 or 5 out of 6) and consistent effect directions. Among this, 7 miRNAs (miR-20a_b, miR-101, miR-152, miR-340, miR-374–5p, miR-409–3p, and miR-590–5p) held beta coefficients above 0, hence reflecting an increased functionality over time with increasing miRNA level, and 18 miRNAs (let7f, miR-10b_b, miR-1274a, miR-138, miR-148a, miR-155, miR-183_b, miR-194, miR-204, miR-323–3p, miR-375, miR-487b, miR-516–3p, miR-520d-5p, miR-532–3p, miR-548a, miR-645, miR-95) held beta coefficients below 0, hence reflecting a decreased functionality over time with increasing miRNA level. Of these miRNAs, miR-10b_b, miR-148a, miR-194, miR-323–3p, and miR-516–3p were negatively associated to five phenotypes, while miR-20a_b and miR-340 were positively associated to five phenotypes. Lastly, miR-135a and miR-151–3p held p values below 0.05 for all 6 phenotypes, yet the beta coefficients did not display the same direction of effect for all phenotypes (Supplementary Table 12).

#### 3.2.2. Pathway analysis of the miRNAs identified in the longitudinal analyses

**Fig. 3.**
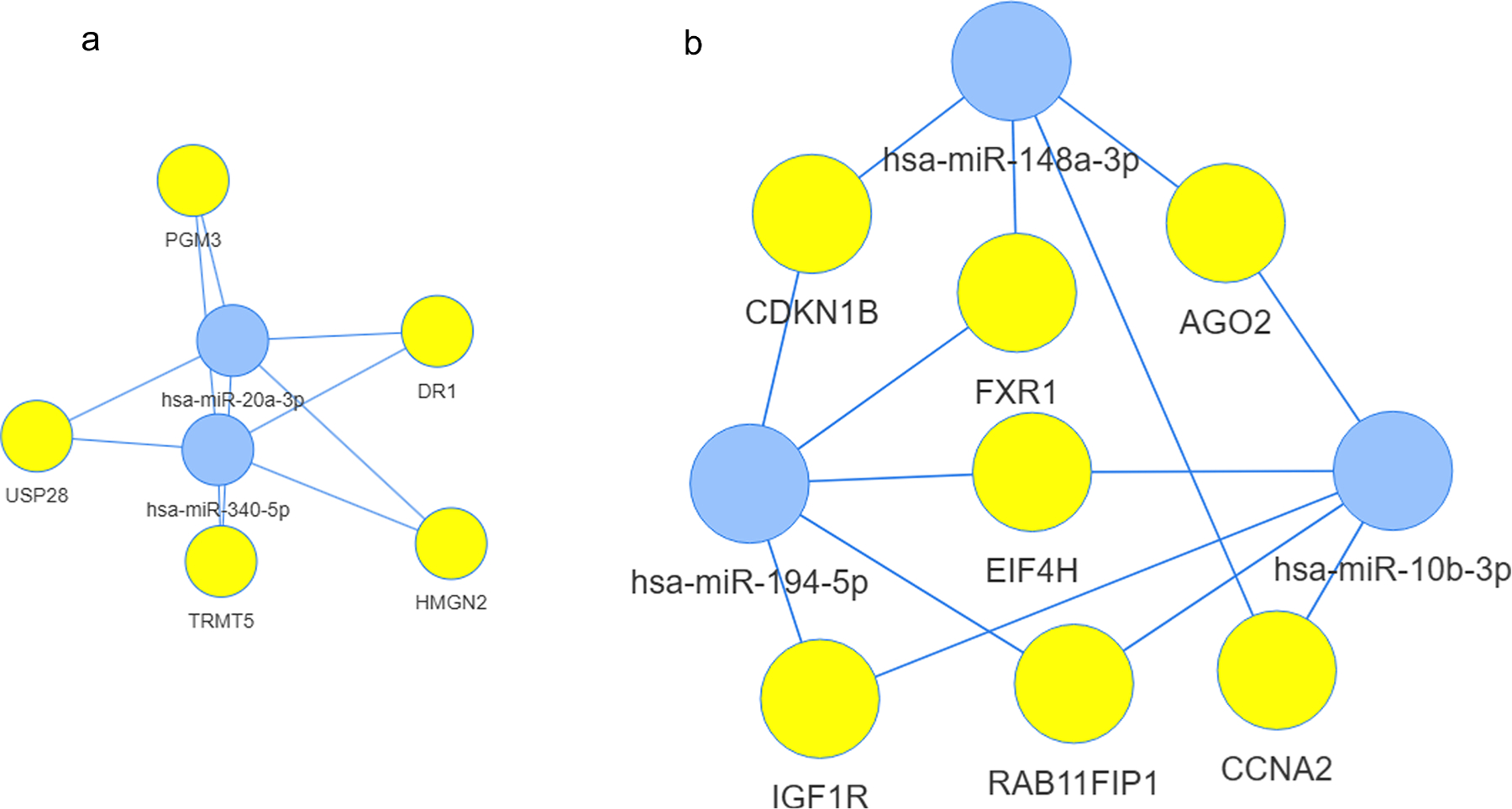
Networks obtained by miRNA-target enrichment analysis (using MIENTURNET) for miRNAs identified in the longitudinal analyses. (a) The network for the two miRNAs found to display a positive beta coefficient in the longitudinal analyses across five phenotypes. (b) The network for the five miRNAs found to display a negative beta coefficient in the longitudinal analyses across five phenotypes.

**Fig. 4.**
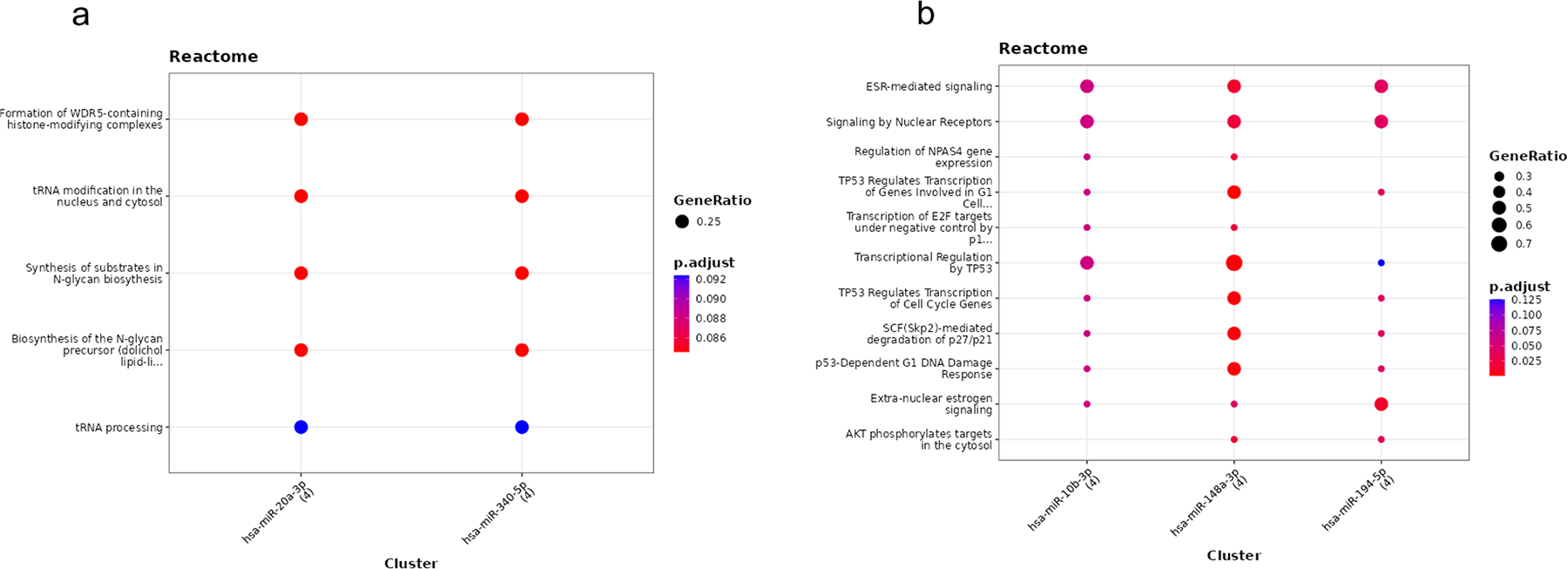
Pathways identified for the miRNAs identified via network analysis (using MIENTURNET) for miRNAs identified in the longitudinal analyses. (a) Pathway analysis by the Reactome database of the two miRNAs found to display a positive beta coefficient in the longitudinal analyses. (b) Pathway analysis by the Reactome database of the three miRNAs found as central hub in the network of the five miRNAs found to display a negative coefficient in the longitudinal analyses. Notes: the dots are scaled by the ratio of enriched miRNA targets to the total genes in each pathway. Dot color reflects the adjusted p-value, enabling simultaneous visualization of enrichment magnitude and statistical significance across categories.

To gain biological insight into the functional relevance of the miRNAs exhibiting a consistent direction of effect across at least five of the six phenotypes, we performed miRNA-target enrichment analysis. We followed the same approach as in the cross-sectional analysis, i.e., we grouped the miRNAs according to their beta values in the association analysis. A total of 5 target genes were identified for the two miRNAs with a positive beta value (i.e., miR-20a_b and miR-340), whereas 7 target genes were detected for the five miRNAs with a negative beta value (i.e., miR-10b_b, miR-148a, miR-194, miR-323–3p, and miR-516–3p) (Supplementary Tables 13 and 14). As depicted in the network analysis in Fig. 3a and REACTOME enrichment analysis (Fig. 4a and Supplementary Table 15), the two most relevant miRNAs showing an increase of functionality with their increased expression (positive beta coefficients) were miR-20a_b and miR-340, and target genes (*USP28*, *TRMT5, PGM3*, *DR1*, *HMGN2*) involved in metabolism of RNA, metabolism of proteins and gene expression, this last through the regulation of histone-modifying complexes. Conversely, as depicted in Fig. 3b (and further documented in Fig. 4b and Supplementary Table 15), the miRNAs associated with a decrease in functionality over time (negative beta coefficients), namely miR-10b-3p, miR-148a-3p and miR-194–5p, target genes like *IGF1R*, *FXR1*, *RAB11FIP1, EIF4H*, *AGO2*, *CCNA2*, *CDKN1B*, associated to signal transduction, regulation of cell cycle and regulation of gene expression, in particular of TP53-regulated transcriptional programs.

### 3.3. Follow-up of the 14 miRNAs from cross-sectional analysis in longitudinal data

Among the 14 miRNAs identified in the cross-sectional analyses (Table 2), comparison with longitudinal results showed that miR-625 and miR-518d retained positive beta coefficients, while miR-339–5p, miR-766, miR-628–5p, miR-1274a, and miR-221 retained negative coefficients (Table 3). Of these, only miR-1274a was statistically significant in both the cross-sectional and the longitudinal analyses, indicating a consistent effect over time. The remaining 7 miRNAs displayed opposite, non-significant effects, except miR-27b, which exhibited significant effects in opposite directions, suggesting its impact may vary over time.

## 4. Discussion

The present study investigated the association between circulating miRNAs and physical functioning and frailty in a cohort of older twins, applying both individual and twin intra-pair analyses, the latter controlling genetic confounding. Large part (i.e., 84 %) of the miRNAs identified at the individual level displayed the same direction of effect at the twin pair level, supporting a role for these miRNAs. In addition to investigation of phenotype data at baseline, longitudinal measurements were explored with follow-up for up to eight years. Overall, more significant findings were observed in this longitudinal analysis, suggesting that miRNAs might be more precise for predicting the decline in functioning over time than the cross-sectional variation at baseline. Finally, most of the effect sizes observed were negative (i.e., 79 % in the cross-sectional analysis, and 65 % in the longitudinal analysis) reflecting a decreased functionality with increased miRNA levels, suggesting that the miRNAs reduce expression of genes collectively being beneficial for functional capacity in old age.

In the cross-sectional analyses, 14 miRNAs displayed association at both individual and twin pair levels: three with positive beta coefficients, and 11 with negative beta coefficients. The literature supports the relevance of a number of these miRNAs for muscle function; for instance, inhibition or overexpression of miR-15b has been shown to promote or inhibit myoblast differentiation, respectively, through a mechanism believed to involve the binding of miR-15b to the 3′ untranslated region of the SET Domain Containing 3, Actin N3 (Tau)-Histidine Methyltransferase (SETD3) (Zhao et al., 2019). Also, miR-628–5p inhibits the transcription of striated muscular activator of rho signaling (STARS) preventing muscle cell regeneration, and in older individuals, acute resistance exercise reduces the expression of miR-628–5p in skeletal muscle, facilitating muscle regeneration (Russell et al., 2017). Lastly, in mice miR-221 is expressed at a lower level in aged muscle tissue (Hamrick et al., 2010). Interestingly, both miR-15b and miR-221 were found as central hubs in the network analysis in the present study.

Nevertheless, in such cross-sectional analysis the temporality is not clear; it cannot be determined whether miRNA levels increase or decrease before or after a change in physical functioning, an aspect, which is addressed in the longitudinal analysis, where miRNA levels in principle predict future decline in function. That more miRNAs were identified in the longitudinal analysis might be expected; studies indicate that the mechanisms associated with disease progression over time might be different from those present at baseline (Guralnik and Kritchevsky, 2010). This is likely particularly relevant in the present study, as the individuals were selected at intake as belonging to complete twin pairs (i.e., both individuals were still alive at a minimum age of 73 years (age range (mean): 73–88 (79)); consequently, the individuals are likely in better health compared to randomly selected singletons. Categorization of frailty at base line revealed only two of the 86 individuals to be frail and using values on hand grip strength and chair stand test to classify individuals as ‘probably sarcopenic’ (as defined by Cruz-Jentoft et al., 2019), only three individuals were categorized as such. Hence, as the variation health is likely small at baseline, as time passes the variation in health is likely to increase, as some individuals will experience a decline. In statistical terms, this implies that certain miRNAs may predict the deterioration of the phenotype over time, but the same miRNAs might not be significantly associated at baseline. Hence, the results of the longitudinal analysis support miRNAs as relevant biomarkers for functional decline during aging. Similarly to the cross-sectional analysis, more negative than positive beta coefficients were observed, suggesting that increased miRNA levels at baseline associate with a subsequent decline in functional capacity. Specifically, seven miRNAs displayed association with the same direction of effects across 5 of the 6 phenotypes: five miRNAs with negative effect sizes and two with positive effect sizes. The literature supports a role in muscle biology of several of these miRNAs; for instance, miR-148a has been shown to regulate Rho Associated Coiled-Coil Containing Protein Kinase 1 (ROCK1), stimulating myogenic differentiation and muscle regeneration and differentiation (Zhang et al., 2012). Also, both acute exercise and chronic resistance have been observed to reduce miR-148a levels (Nielsen et al., 2014). Increased levels of miR-194–5p have been found in frail patients (Ipson et al., 2018) and an involvement of miR-194–5p in cellular senescence, generation of reactive oxygen species, and modulation of muscle homeostasis (e.g., Shi et al., 2021) support such association. Similarly, miR-20a is downregulated during ageing (Hackl et al., 2010), and promotes myoblast differentiation, and suppresses proliferation (Luo et al., 2016). Finally, miR-340–5p is highly expressed in cellular senescence, and as its overexpression leads to sensitivity to senolytics (causing apoptosis), miR-340–5p is a suggested anti-aging target (Herman et al., 2021). Interestingly all these miRNAs were found as central hubs in the network analysis of the present study.

Finally, inspection of the 14 miRNAs found in the cross-sectional analyses in the longitudinal data revealed miR-1274a to hold significant negative association in both analyses for the ADL endurance score. Also, the direction of effect was the same in 17 out of the 18 analyses performed for miR-1274a, although not all were statistically significant. While studies have indicated a role of miR-1274a in cancer (e.g., Ren et al., 2020), Alzheimer’s disease (Denk et al., 2015), diabetes (Collares et al., 2013) and respiratory disease (Gon et al., 2020), a role in muscle biology has, to the best of our knowledge, not been reported. However, one target of miR-1274a is Forkhead Box O4 (FOXO4) (Wang et al., 2015), which is highly expressed in the skeletal and cardiac muscle (Chen et al., 2022). However, according to bioinformatic analyses miR-1274a is currently not classified as a canonical microRNA, but rather as a fragment derived from a tRNA, specifically tRNA-Lys (Schopman et al., 2010, Desvignes et al., 2015). Nevertheless, such tRNA-derived fragments (tDRs), also known as tRNA-derived small RNAs (tsRNAs), belong to the category of non-coding RNAs with structural and functional properties comparable to those of microRNAs and recent studies have shown that tDRs can play regulatory roles in translation, mRNA stability and biological processes (Zhao et al., 2023; Bakowska-Zywicka et al., 2025). Therefore, further research into 1274a is very much warranted.

Lastly, by splitting the miRNAs identified in the association analyses by their direction of effects, i.e., negative (higher miRNA expression associated with worse performance) or positive (higher miRNA expression associated with better performance), generating networks and performing pathway analyses, the molecular pathways targeted by the miRNAs were explored. Overall, the miRNAs posing a negative effect affected genes involved in foremost gene expression (29 %), the immune system (21 %), and signal transduction (21 %), yet also the cell cycle (11 %), developmental biology (7 %), programmed cell death (7 %), and hemostasis (4 %). The miRNAs with a positive effect, also affected genes related to signal transduction (29 %) and the immune system (29 %), yet to a lesser extend gene expression (7 %), however also metabolism of proteins (14 %) and metabolism of RNA (29 %), the latter related to mRNA decay and tRNA processing. Interestingly, the immune pathways identified were somehow different; the miRNAs with a positive effect foremost affected pathways related to interferon function, while the miRNAs with a negative effect affected both interferons and interleukins, hence presumably reflecting immune responses to virus infection, respectively, a more regulatory effect on the immune system. Immune pathways are key for the aging process; low-grade inflammation has been reported for older individuals, and chronic subclinical inflammation may be a marker of functional limitations, e.g., have elevated levels of inflammatory biomarkers been reported to associate to low performance in hand grip strength and chair stands tests (Brinkley et al., 2009; Ji et al., 2022). Regarding signal transduction, miRNAs with positive effect affected genes related to Rho Related BTB Domain Containing (RHOBTB) signaling, while the miRNAs with negative effects affected pathways related to among others hormone signaling, including estrogen signaling. The estrogen pathways included both within and extra-nuclear effects, where the former affects among others cellular proliferation and differentiation, hereby affecting e.g., reproduction, metabolism, and neuronal and cardiovascular functions, while the latter affects among others apoptosis, cellular proliferation, and neural function (Arnal et al., 2017). RHOBTB represents a family of proteins acting as tumor-suppressors, yet also with an effect on regulation vascular function and blood pressure (Wei and Rivero, 2016), phenotypes, which in themselves are key for physical and functional capacity in old age. Lastly, the gene expression pathways seen for the miRNAs with a negative effect pointed to two central regulators of gene transcription: p53 and the FOXO transcription factors. p53 affects a wide array of processes, including DNA repair, apoptosis and cellular senescence, all processes of relevance to aging, yet p53 has also been linked to exercise metabolism (Bartlett et al., 2014). The specific pathways found here for p53 related to regulation of the cell cycle, possibly reflecting an effect in muscle regeneration. A role of the FOXO factors is very appealing, as several studies have demonstrated their role in the regulation and preservation of skeletal muscle homeostasis, controlling important processes such as energy metabolism, autophagy, and the reaction to oxidative stress (e.g., Chen et al., 2022). Also, FOXO proteins have been linked to ageing and long life through interactions with insulin and the mTOR pathways (e.g., Du and Zheng, 2021), a relevant observation considering the age range of the present cohort (73–88 years).

Finally, the present study has several strengths; first of all, to the best of our knowledge, this study is the most comprehensive study to date regarding miRNA and phenotypes of physical and functional capacity. Secondly, the use of twins enables the investigation of discordant twins, hence reducing confounding induced by variation in genetics and early life environment, and the inclusion of longitudinal data enables investigation of decline in functioning over time. One limitation of the present study is, however, that the miRNA values were based on blood samples, which means that the values reflect the overall biology of an individual, and not the biology specific to for instance a muscle cell. However, molecular biomarkers measured in blood are clinically relevant due to the ease of obtaining a blood sample compared to a more invasive muscle tissue biopsy. Secondly, it is broadly recognized that there is a difference in age-related physical decline between sexes (e.g., Huebner et al., 2022), however, this aspect could not be comprehensively investigated in the present study due to the distribution of males (N = 20) and females (N = 66). Thirdly, as mentioned above, as the individuals of the present study were selected as complete twin pairs, they are likely in better health compared to randomly selected singletons. This was also reflected in the few frail individuals of the present study. Hence, the findings of the present study might not be directly generalized to a general, and potentially less healthy, population of septuagenarians and octonaries. Fourthly, we did not perform correction for multiple testing in the present study, as we perform an explorative study of phenotypes, which in general are not all well studies in the literature. Yet, the discordant twin pair design is statistically powerful, needing down to only one-tenth the number of individuals compared to the classical case control study (Li et al., 2018). Nevertheless, replication studies are warranted to confirm our findings.

In conclusion, the present study points to several miRNAs as associated to physical functioning and functional capacity and decline as people age, many of these exhibiting increased expression in relation to poorer function. miRNAs like miR-15b and miR-221, central hubs in our network analysis, could serve as potential targets for future investigations. Moreover, our findings show that miR-1274a is associated with physical endurance at both the baseline and over time, suggesting that it could be considered a potential biomarker of physical decline.

## Supplementary Material

1

## Figures and Tables

**Table 1:** Characteristics of the study population.

Variables	
No. individuals	86
No. monozygotic twin pairs	43
Age (years), mean ± SD, range	79.05 (3.51), 73–88
Women, N (%)	66 (76.74 %)
BMI (kg/m^2^), mean ± SD	23.5 (3.81)
Height (cm), mean ± SD	164.5 (7.50)
Hand grip strength (kg)*, N, mean ± SD, no. discordant: concordant twin pairs	70, 22.44 (7.23), 29:2
Chair stand (sec)*, N, mean ± SD, no. discordant: concordant twin pairs	65, 11.91 (3.09), 27:0
ADL fatigue, N, mean ± SD, no. discordant: concordant twin pairs	86, 5.34 (1.25), 21:22
ADL strength, N, mean ± SD, no. discordant: concordant twin pairs	86, 1.55 (0.53), 41:2
ADL endurance*, N, mean ± SD, no. discordant: concordant twin pairs	69, 3.77 (1.06), 28:2
Frailty index, N, mean ± SD, no. discordant: concordant twin pairs	86, 0.07 (0.06), 40:3
Frailty index categories, n (%):	
Non-frail (≤0.1)	70 (81.39 %)
Pre-frail (>0.1 – ≤0.21)#	14 (16.28 %)
Frail (>0.21)#	2 (2.33 %)

Notes: No.: number of, SD: standard deviation, BMI: body mass index, kg: kilo grams, m: meters, cm: centimeters, sec: seconds, ADL: activity of daily living, N: number of individuals with phenotype data.

* These phenotypes were measured in the 1999 wave, whereas the other phenotypes were measured in the 1997 wave (i.e., at the time of the blood sampling).

#: using a cut-off of 0.21 (as defined by Kim and Rockwood, 2024) four individuals were found to be frail and 12 individuals were pre-frail.

**Table 2:** MiRNAs significantly associated with (A) Hand grip strength, (B) Chair stand test, (C) Activity of daily living and (D) Frailty index.

	Individual level analysis					Twin pair level analysis				
miRNAs	no. individuals	Coef	SE	95 % CI	P	no. twin pairs	Coef	SE	95 % CI	P
**(A) Hand grip strength (log transformed)**										
mir625	38	0.128	(0.052)	[0.021; 0.235]	0.021	9	0.444	(0.168)	[0.033; 0.855]	0.038
**(B) Chair stand test (reversed)**										
mir27b	61	−4.881	(1.777)	[−8.488; −1.273]	0.009	25	−5.872	(2.657)	[−11.382; −0.362]	0.038
mir339_5p	24	−2.712	(1.256)	[−5.389; −0.034]	0.047	8	−12.522	(4.591)	[−24.323; −0.721]	0.041
**(C) Activity of daily living (ADL)**										
ADL fatigue score										
mir30b	85	−1.150	(0.364)	[−1.885; −0.415]	0.003	21	−2.330	(0.892)	[−4.135; −0.526]	0.013
mir628_5p	31	−0.610	(0.270)	[−1.170; −0.050]	0.034	5	−2.127	(0.601)	[−3.670; −0.583]	0.017
ADL strength score (reversed)										
mir766	85	−0.231	(0.082)	[−0.397; −0.065]	0.007	40	−0.340	(0.167)	[−0.677; −0.002]	0.048
mir454	68	0.245	(0.104)	[0.035; 0.455]	0.023	26	0.428	(0.195)	[0.026; 0.830]	0.038
mir15b	85	−0.334	(0.156)	[−0.648; −0.020]	0.038	40	−0.807	(0.321)	[−1.456; −0.158]	0.016
mir518d	77	0.180	(0.089)	[0.001; 0.360]	0.049	34	0.494	(0.181)	[0.125; 0.863]	0.010
ADL endurance score (reversed)										
mir601	55	−0.452	(0.121)	[−0.698; −0.207]	0.001	34	−0.891	(0.369)	[−1.683; −0.098]	0.030
mir1274a	68	−0.611	(0.188)	[−0.991; −0.230]	0.002	54	−1.181	(0.354)	[−1.909; −0.454]	0.003
mir720	68	−0.555	(0.228)	[−1.017; −0.093]	0.020	54	−0.774	(0.358)	[−1.509; −0.038]	0.040
**(D) Frailty index (reversed)**										
mir221	85	−0.050	(0.021)	[−0.097; −0.013]	0.011	39	−0.070	(0.033)	[−0.134; −0.002]	0.045
mir423_5p	85	−0.040	(0.020)	[−0.085; −0.003]	0.036	39	−0.070	(0.024)	[−0.115; −0.018]	0.008

Notes: no.: number of, Coef: beta coefficient, SE: standard error, 95 % CI: 95 % confidence interval, p: p value.

**Table 3:** Comparison of the miRNAs that demonstrated a significant correlation in the individual analysis across the phenotypes versus the longitudinal analysis.

Individual Analysis						Longitudinal Analysis				
**Hand grip strength (log transformed)**										
miRNAs	N	Coef	SE.	95 % CI	p	N	Coef	SE	95 % CI	p-value
mir625	38	0.13	0.05	[0.02;0.24	0.020	27	0.01	0.04	[−0.09; 0.08]	0.97
**Chair stand test (reversed)**										
mir27b	61	−4.88	1.78	[−8.49; −1.27]	0.009	41	0.48	0.24	[0.02; 0.94]	0.042
mir339_5p	24	−2.71	1.26	[−5.39; −0.03]	0.047	20	−0.13	0.27	[−0.66; 0.39]	0.614
**ADL strength score (reversed)**										
mir766	85	−0.23	0.08	[−0.40; −0.07]	0.007	71	−0.01	0.02	[−0.05; 0.03]	0.639
mir454	68	0.25	0.10	[0.04; 0.46]	0.023	57	−0.00	0.02	[−0.04; 0.04]	0.833
mir15b	85	−0.33	0.16	[−0.65; −0.02]	0.038	72	0.04	0.06	[−0.08; 0.16]	0.518
mir518d	77	0.18	0.09	[0.01; 0.36]	0.049	65	0.01	0.01	[−0.01; 0.02]	0.124
**ADL fatigue score**										
mir30b	85	−1.15	0.36	[−1.89; −0.42]	0.003	71	0.01	0.07	[−0.12; 0.14]	0.897
mir628_5p	31	−0.61	0.27	[−117; −0.05]	0.034	25	−0.01	0.14	[−0.28; 0.26]	0.942
**ADL endurance score (reversed)**										
mir601	55	−0.45	0.12	[−0.70; −0.21]	0001	44	0.01	0.05	[−0.09; 0.10]	0.889
mir1274a	68	−0.61	0.19	[−0.99; −0.23]	0.002	53	−0.06	0.01	[−0.08; −0.05]	1.4*10^−13^
mir720	68	−0.55	0.23	[−102; −0.09]	0.020	52	0.02	0.04	[−0.05; 0.10]	0.559
**Frailty index (reversed)**										
mir221	85	−0.05	0.02	[−0.10; −0.01]	0.011	72	−0.01	0.01	[−0.02; 0.02]	0.945
mir423_5p	85	−0.04	0.02	[−0.09; −0.01]	0.036	71	0.01	0.01	[−0.01; 0.01]	0.844

Notes: N: number of individuals, Coef: beta coefficient, SE: standard error, 95 % CI: 95 % confidence interval, p: p value.

## Data Availability

Due to Danish and EU legislations, data sharing requires case-by-case approval from the Danish Data Protection Agency. Hence, the data are not deposited in a public database. We welcome any inquiries.

## Appendix A. Supporting information

Supplementary data associated with this article can be found in the online version at doi:10.1016/j.mad.2025.112099.

## Abbreviations:

BMI: body mass index
cm: centimeters
FDR: false discovery rate
FOXO: Forkhead Box O
kg: kilograms
LSADT: Longitudinal Study of Aging Danish Twins
m: meters
miRNAs: MicroRNAs
mRNAs: messenger RNAs
MZ: monozygotic
qRT-PCR: quantitative real-time PCR
ROCK1: the Rho Associated Coiled-Coil Containing Protein Kinase 1
SE: standard error
sec: seconds
SETD3: the SET Domain Containing 3, Actin N3Tau-Histidine Methyltransferase
SD: standard deviation
STARS: the striated muscular activator of rho signaling
